# Effect of Stem Cell Therapy on Patients’ Quality of Life in Heart Failure with Reduced Ejection Fraction

**DOI:** 10.25122/jml-2018-0013

**Published:** 2018

**Authors:** Zahra Mostafavian, Farveh Vakilian, Leila Torkmanzade, Toktam Moghiman

**Affiliations:** 1. Department of Community Medicine, Mashhad Branch, Islamic Azad University, Mashhad, Iran; 2. Cardiothoracic Research Center, Faculty of Medicine, Mashhad University of Medical Sciences, Mashhad, Iran; 3. Faculty of Medicine, Islamic Azad University, Mashhad Branch, Mashhad, Iran; 4. Mashhad University of Medical Sciences, Mashhad, Iran

**Keywords:** Stem cell, Quality of life, Heart Failure

## Abstract

**Introduction:** Heart failure with reduced ejection fraction (HFrEF) is a debilitating disease in which Left Ventricular Ejection Fraction (LVEF) is ≤ 40%, and it involves various organs. Regarding the novelty of stem cell therapy in HF, we aimed at studying the effect of stem cell therapy on the QoL of patients with HFrEF.

**Materials and Methods:** In a prospective study, 30 patients diagnosed with HFrEF who had undergone stem cell injection (study group) and 30 patients with HFrEF receiving guideline-directed medical therapy (control group) were recruited by convenience sampling during 2016 in Mashhad, Iran. Patients’ quality of life, left ventricular ejection fraction and their disability degree were studied twice with a 3-month interval. For data analysis, paired t-test, chi2 and multivariate linear regression were used.

**Results:** The mean age of study and control groups was 61.3±10.24 and 60.93±7.88 years respectively. Ninety percent of the cases and 56.7% of the controls were male (P=0.003). A significant difference in QoL was observed before and after treatment in each group (P<0.05). However, the QoL score showed no statistical difference between the two groups following treatment (P=0.13). The same result was achieved for LVEF (P=0.18); whereas the NYHA function class showed a significant difference between the two groups following treatment (P=0.017).

**Conclusions:** According to the results, it seems that the treatment of HFrEF patients with stem cells is as effective as conventional therapies in improving the LVEF and QoL and more efficient than conventional treatments in increasing the patients’ general satisfaction with life.

## Background

Variable classifications have been introduced for heart failure (HF) based on the severity of clinical symptoms, clinical stages or ejection fraction. Heart failure with reduced ejection fraction (HFrEF) is a debilitating disease in which Left Ventricular Ejection Fraction (LVEF) is ≤ 40% and involves various organs resulting in increased mortality rates or severe clinical disability in the patient [1]. Therefore, it has a significant impact on the patient’s quality of life (QoL) and survival, especially in elderly patients [2]. Different etiologies have been mentioned for the incidence of HF, most importantly myocardial infarction and coronary artery disease [3]. Over the years numerous attempts have been made to discover medical and non-medical treatment options for this condition. Medical treatments have in some cases led to increased survival and better QoL, besides the increased tendency to non-medical therapeutic options. Today, heart transplantation can be a peculiar option for end-stage HFrEF, but it has many complications and is not eligible for all patients. Ten years ago, the administration of stem cells was introduced as a therapeutic alternative for HF patients, especially for those cases resulting from cardiac ischemia or infarction [4, 5].

Stem cells are non-differentiated cells which can turn into active and effective cells in specific tissues and have beneficial effects on tissue repair. The application of stem cells has been mentioned in different diseases, and their role as a scaffold in the healing of cardiac tissue or their paracrine effects in cell apoptosis prevention has been highlighted [6,7].

Numerous studies have investigated the role of stem cells on cardiac function improvement and tissue repair; however, their role in clinical improvement or QoL has been less commonly evaluated [8–10].

Regarding the novelty of the stem cell injection therapeutic method, this study is aimed at investigating the QoL in patients with heart failure before and after the infusion of stem cells and its comparison with the control group.

## Materials and Methods

### Study design and population

This study was a prospective group design. The study population consisted of 30 consecutive HF outpatients with a LVEF ≤ 40% at optimal medical treatment at the time of inclusion, who were referred to the Cardiology clinic of the Medical School of the University of Mashhad, Iran, during the year 2016, in order to receive stem cell therapy in addition to the conventional treatments. The decision for stem cell therapy was made according to the patients’ will in addition to their physicians’ decision. Also, 30 patients with HF and (LVEF) ≤ 40% who were referred to the same clinic at the same time for receiving conventional treatments according to guideline-directed medical therapy (GDMT) were recruited by convenience sampling. All the patients were on full tolerable medical treatments for over three months which include Lisinopril 5 bid, Varvedilol 12.5 bid, Lasix 40 bid and Aldactone 25 qd at least. All drugs used in our study were made in Dr. Abidi Pharmaceutical Laboratory in Iran. Patients enrolled in the study were over 18 years of age, at least three months away from the initial diagnosis of the disease, having the ability to communicate and complete the questionnaire besides having consented to participation in the trial. Patients were excluded from the study if their serum creatinine level was >2.5mg/dl, increased liver enzymes to over three times the average level, the presence of life-threatening arrhythmias and congenital cardiac disease and history of a known psychiatric disorder or psychiatric/psychological treatment.

### Stem cell therapy

The patients who were in the group of stem cell therapy underwent bone marrow aspiration in Montaserieh Hospital of Mashhad, Iran. One hundred millimeter fluid was aspirated from their bone marrow and stored in a cool box; it was sent to the Royan Institute in Tehran via air. In the Royan Institute, the samples were precisely examined for a blood infection, and the mononuclear cells of the bone marrow were extracted by centrifugation. The samples were then returned to Mashhad. On the first morning after receiving the samples, the patients underwent coronary angiography, and 5cc cells consisting of 6 to 8 million mononuclear cells were injected via a microcatheter in the proximal part of the injured coronary artery (which was previously determined by cardiac scan or echocardiography). If no complication occurred, the patients were discharged and follow-ups were done one week and three months later. The researching cardiologist attended all stages of the stem cell infusion and was not blinded for the patients’ group.

### Measurements

The quality of life, left ventricular ejection fraction and the disability degree were studied in two groups of patients. LVEF was determined by echocardiography, and the patients’ disability level was determined using the New York heart association functional classification (NYHA FC). All measurements were done by the researching cardiologist twice at a 3-month interval. That way, in the group of stem cell therapy measurements, were done before the injection of stem cells and three months later, and in the control group at the beginning of the study and three months later.

### Quality of life measurements

The IHF-QoL was used to assess QoL in patients. It has 15 questions and studies 5 domains of QoL including the symptoms and their severity, physical limitations, social interference, psychological condition and self-efficacy, and knowledge. Each field has 5, 1, 4, 3 and 2 questions, respectively. Question number 16 is a conclusive item in which the patients respond to their overall quality of life as unfavorable, moderately favorable, and favorable. The questionnaire was developed and validated by Naderi et al. in 2013 in Iran [11].

### Ethics

The study protocol was approved by the Ethics Committee of The Islamic Azad University of Mashhad with this code (IR.IAU.MSHD.REC.1396.115). The patients filled in a written informed consent prior to study entry. The questionnaires were anonymous, and all collected data were regarded as confidential.

### Statistical analysis

The Statistical Package for the Social Sciences (SPSS Statistics 17.0) software was used to analyze the data. All continuous variables are expressed as mean ± SD and for some categorical as numbers and percentages. All variables were tested for normal distribution. Paired samples t-test was used to compare quantitative variables before and after treatment in each group, and independent samples t-test was used for intergroup comparisons. Qualitative variables were compared with the chi-square test. A multivariate linear regression analysis using a simultaneous entry of predictors was performed to examine which variables were correlated independently to QoL scores. A p-value of 0.05 or lower was considered statistically significant.

## Results

In total 60 cases with HF were studied in either the study or control group. The patients’ demographic data are presented in Table 1. Except for sex distribution which showed a significant difference between the two groups (P=0.003), none of the other variables were significantly different in this respect.

Table 2 summarizes the baseline and follow-up variables for the study and control groups. As shown in this table, the QoL in patients in the stem cell therapy group improved significantly three months after receiving the treatment. The questionnaire score increased from 41±7.6 before treatment to 53.13±5.33 three months after treatment (P=0.001). The LVEF also showed a meaningful difference in these patients rising from 24.77±6.35 (before treatment) to 31.3±5.09 three months after receiving the treatment (P=0.001). Moreover, the NYHA function class showed a significant improvement in reducing from 3±0.83 to 2.4±0.67 during the same period (P=0.002).

Nevertheless, in the group receiving conventional therapy, improvement in the QoL, NYHA function class and LVEF was also observed. As of three months after the initiation of conventional therapy, the QoL score changed from 39±8.4 to 55.3±4.45 (P=0.001); the NYHA function class reduced from 3.36±0.8 to 1.87±0.97 (P=0.001) and the LVEF increased from 24.7±8.9 to 29.3±6.8 (P=0.001).

The QoL score showed no meaningful difference between the two groups following treatment (P=0.13). The same result was achieved for LVEF (P=0.18), whereas the NYHA function class showed a significant difference between the two groups following treatment (P=0.017). That way, the control group had a better situation in this regard.

Given the last question of the IHF-QoL questionnaire, which is a conclusive question regarding the patient’s general satisfaction with his/her life quality, the two groups were compared before and after the treatment.

As demonstrated in Table 3, no meaningful difference was observed between the two studied groups regarding the general satisfaction of life before treatment (P=0.21), whereas a statistically significant difference was achieved after treatment (P=0.02).

In order to study the independent effect of each variable including treatment group, age, sex, marital status, and the quality of life before treatment on the post-treatment quality of life, all variables were entered into the multivariate linear regression model.

**Table 1:** Clinical characteristics of the patients in the two studied groups (at the beginning of the study).

	Study group (n=30)	Control group (n=30)	P-value
Age (yrs)	61.3±10.24	60.93±7.88	0.87
Male	27 (90%)	17(56.7%)	0.003
Married	28(93.3%)	30(100%)	0.49
QoL scores	41±7.6	39±8.4	0.35
NYHA FC	3±0.83	3.36±0.8	0.089
LVEF (%)	24.77±6.35	24.7±8.9	0.97

**QOL:** quality of life, **NYHA FC:** New York heart association functional classification, **LVEF:** left ventricle ejection fraction

**Table 2:** Comparison of baseline and follow up variables for the study and control group.

	Baseline		p-value	3 months after treatment initiation		p-value
	Study (n=30)	Control (n=30)		Study (n=30)	Control (n=30)	
QoL scores	41±7.6	39±8.4	0.350	53.13±5.33	55.3±4.45	0.130
NYHA FC	3±0.83	3.36±0.80	0.089	2.4±0.67	1.86±0.97	0.017
LVEF%	24.77±6.35	24.7±8.9	0.975	31.3±5.09	29.3±6.8	0.182

**QOL:** quality of life, **NYHA FC:** New York heart association functional classification, **LVEF:** left ventricle ejection fraction

**Table 3:** Univariable analysis of stem cell injection effect on general satisfaction.

General satisfaction	Before treatment				3 months after treatment			
	Stem cell therapy		Conventional therapy		Stem cell therapy		Conventional therapy	
	No	%	No	%	No	%	No	%
Unsatisfied	19	63.3	0	0	0	0	2	6.7
Occasionally satisfied	11	36.7	8	26.7	8	26.7	16	53.3
Satisfied	0	0	22	73.3	22	73.3	12	40
Total	30	100	30	100	30	100	30	100
P-value	P= 0.21				P=0.02			

Solely the QoL before treatment and sex had a significant impact on the post-treatment QoL; this way, the better the pre-treatment QoL, the better the post-treatment QoL (p=0.015). Moreover, QoL achieved a higher score in males in comparison to females (p=0.023). The conclusion that men do respond better to the treatment can be drawn from these results.

## Discussion

In the present study, cardiac function, clinical condition and QoL of patients with chronic HF and reduced EF were studied and compared in two group – medical therapy and medical therapy plus stem cell therapy. No meaningful difference was observed in the demographic characteristics and EF between the two groups except for sex at the beginning of the study. Patients’ QoL increased significantly after treatment and to a similar level in both groups. Regarding participants’ sex, QoL improvement was higher among males. Given the patients’ general satisfaction with life, there was no satisfaction in either group before treatment; whereas, after completing the treatment course, general satisfaction of life was significantly higher in the group receiving stem cell therapy.

Regarding stem cell therapies in the treatment of HF, few studies have been conducted in Iran. Mohyeddin-Bonab et al. conducted one study in Iran with the title of “Autologous in Vitro Expanded Mesenchymal Stem Cell Therapy for Human Old Myocardial Infarction” on 8 patients in a study group and 8 patients in a control group. The results of this study showed Left Ventricular Ejection Fraction increased significantly in the test group (P= 0.005), but not in the control group. In comparison, between the test and control groups, the results of NYHA assessment demonstrated significant improvement in the test group. The reason for the difference between the results of their study and ours is the type of patients studied. In Mohyeddin-Bonab et al.’s study, all 8 patients had an old myocardial infarction. However, in our study, patients with heart failure had different conditions [12].

Randomized clinical trials with autologous and allogeneic mesenchymal stem cells (MSCs) have reported differing results regarding the evolution of left ventricular systolic function and patients’ QoL. [13–18]. The Congestive Heart Failure Cardiopoietic Regenerative Therapy (CHART-1) trial aimed to validate cardiopoiesis-based biotherapy in a larger heart failure cohort. Conducted by Bartunek et al., the study showed neutral results regarding composite, including all-cause mortality, worsening heart failure events, and surrogate endpoints like LVEF in HFrEF patients with ischemic cardiomyopathy receiving intramyocardial injections of cardiopoietic cells (MSC; n=120) versus sham procedures (n=151) [19].

In Mathiasen et al.’s study, a randomized, double-blind, placebo-controlled trial, patients with severe ischemic heart failure were randomized 2:1 to intra-myocardial injections of MSC or placebo, respectively. Like in our study, no differences were found in the QoL that was measured by the Kansas City Cardiomyopathy Questionnaire between the study group and placebo. However, unlike in our study, there were significant improvements in LVEF of 6.2% in the study group compared with the placebo and no differences were found in NYHA class [20].

In 2011, Prin et al. investigated the efficiency of autologous bone marrow mononuclear cells injection in improving the cellular function of patients with ischemic HF. The angina score and tissue perfusion in the group treated with stem cells improved significantly, and regarding age, the therapeutic response was significantly higher in younger patients. In addition, the 6-month QoL score showed a significant improvement in comparison to the medical therapy group [21]. The findings of this study correlated in satisfaction but not in the QoL; this could be due to the methodology, cases and controls characteristics, evaluation methods and the questionnaire type. The control group in Prin et al.’s study consisted of only 10 patients, and the study course was longer. It seems that the highest number of stem cells in different studies was recorded 6 months to 2 years after the injection and therefore our study required a more extended follow-up period.

In the study done by Bartonak et al. in 2013, cardiac EF and size improved significantly. Moreover, clinical parameters such as QoL and event-free survival also improved. The type of injected cells was cardiopoietic, with which a better therapeutic response is seen in treatment, considering their specificity. Their findings on QoL were similar to ours, despite the difference in the questionnaire type and the 6-month follow-up period [22]. Lunde et al. studied the activity capacity and QoL in 50 patients with myocardial infection following stem cell injection in 2007; activity capacity and oxygen consumption were significantly higher in the stem cell group compared to the control group [23].

Although the QoL did not differ in this study, the inconsistency of the results is owed to the two different studied groups. The MI patients do not experience QoL reduction as the HF patients do. They usually return to their previous QoL one month after treatment initiation; whereas chronic HF, due to its extensive effects on different body organs, severely affects the QoL. Accordingly, non-medical factors might strengthen and support the QoL of patients [24].

In Bartolucci’s study that aimed to evaluate the safety and efficacy of the infusion of umbilical cord-derived mesenchymal stem cells (UC-MSC) in patients with chronic stable HFrEF, it was concluded that improvements in left ventricular function, functional status and quality of life in patients with HFrEF were observed in those treated with (UC-MSC) in comparison with placebo. The results of this study are different from our study, and the reason for this is the type of stem cell used in the treatment of patients. As mentioned, in Bartolucci’s study, the type of stem cell used originated in the umbilical cord and our study, it originated in the bone marrow [25].

Taken together, stem cell therapy, regarding its scaffold and paracrine effects can result in prevention of remodeling and HF improvement in ischemic heart cases. This has an important role in improving the symptoms and QoL of such patients [26]. Moreover, better patient follow-up and better and more precise control and regulation of drug therapy and daily regimen during the treatment course can have remarkable outcomes on improving adherence and satisfaction among patients.

### Study limitations

Due to the limitations of expenditure and uncommon use of stem cell therapy as a standard and well-known therapeutic approach, it was not possible to increase the study population, which could by itself affect the assurance of the results, statistical tests and our conclusions regarding the treatment efficacy.

Given the increased risk of patient noncompliance in an elongated follow-up, the patients were only followed-up for three months. However, it seems that with a longer follow-up of patients, a better judgment on the efficacy and safety of treatment could be made.

Due to the limitation in the sample size, it was not possible to select a particular type of HF (e.g., ischemic or non-ischemic), which regarding the different mechanisms of injury, the study would have benefited from.

Nevertheless, future studies are highly recommended to be performed on a larger population, with a longer follow-up (at least 6 months for treatment evaluation and 2 years for assuring the safety of stem cell infusion), and the different types of HF can be studied separately.

## Conclusion

The findings of the current study showed that HFrEF treatment with stem cell injection has equal efficacy to conventional treatments in improving ejection fraction and QoL and a higher efficacy in increasing the patient’s general satisfaction with life. However, the disability degree was lower in those receiving conventional therapy than the stem cell therapy group.

## Acknowledgment

The authors would like to thank the study participants and coordinators, the medical and paramedical staff at the participating institutions.

## Conflict of Interest

The authors confirm that there are no conflicts of interest.

## References

[1] Roger VL, Weston SA, Redfield MM, Hellermann-Homan JP, Killian J, Yawn BP (2004). Trends in heart failure incidence and survival in a community-based population. *Jama*.

[2] Curtis LH, Whellan DJ, Hammill BG, Hernandez AF, Anstrom KJ, Shea AM (2008). Incidence and prevalence of heart failure in elderly persons, 1994-2003. *Arch Intern Med.*.

[3] Colucci W, Braunwald E., Braunwald E (1977). Pathophysiology of Heart Failure.

[4] Yu Hong, Lu Kai, Zhu Jinyun, Wang Jian’an (2017). Stem cell therapy for ischemic heart diseases. *British Medical Bulletin*.

[5] Samadikuchaksaraei A (2006). Stem cell therapy for acute myocardial infarction. *Hellenic J Cardiol*.

[6] de Jong R, Houtgraaf JH, Samiei S, Boersma E, Duckers HJ (2014). Intracoronary stem cell infusion after acute myocardial infarction: a meta-analysis and update on clinical trials. *Circ Cardiovasc Interv.*.

[7] Malani PN (2012). Harrison’s principles of internal medicine. *JAMA*.

[8] Hojjat M, Dastpak M (2010). Stem cells roll in heart disease treatment. *IJCCN*.

[9] Hamano K, Nishida M, Hirata K, Mikamo A, Li T-S, Harada M (2001). Local implantation of autologous bone marrow cells for therapeutic angiogenesis in patients with ischemic heart disease. *Jpn circ J*..

[10] Ozbaran M, Omay SB, Nalbantgil S, Kultursay H, Kumanlioglu K, Nart D (2004). Autologous peripheral stem cell transplantation in patients with congestive heart failure due to ischemic heart disease. *Eur J Cardiothorac Surg*.

[11] Naderi N, Bakhshandeh H, Amin A, Taghavi S, Dadashi M, Maleki M (2012). Development and validation of the first Iranian questionnaire to assess quality of life in patients with heart failure: IHF-QoL. *Research in Cardiovascular Medicine*.

[12] Mohyeddin-Bonab M, Mohamad-Hassani MR, Alimoghaddam K (2007). Autologous in vitro expanded mesenchymal stem cell therapy for human old myocardial infarction. *Arch Iran Med*.

[13] Butler J, Epstein SE, Greene SJ (2017). Intravenous Allogeneic Mesenchymal Stem Cells for Non-Ischemic Cardiomyopathy: Safety and Efficacy Results of a Phase II-A Randomized Trial. *Circ Res.*.

[14] Hare JM, Fishman JE, Gerstenblith G (2012). Comparison of Allogeneic vs Autologous Bone Marrow–Derived Mesenchymal Stem Cells Delivered by Transendocardial Injection in Patients With Ischemic Cardiomyopathy. *JAMA*.

[15] Heldman AW, DiFede DL, Fishman JE (2014). Transendocardial mesenchymal stem cells and mononuclear bone marrow cells for ischemic cardiomyopathy: the TAC-HFT randomized trial. *JAMA*.

[16] Zhao XF, Xu Y, Zhu ZY, Gao CY, Shi YN (2015). Clinical observation of umbilical cord mesenchymal stem cell treatment of severe systolic heart failure. *Genet Mol Res*.

[17] Hare JM, DiFede DL, Castellanos AM (2017). Randomized Comparison of Allogeneic Vs. Autologous Mesenchymal Stem Cells for Non-lschemic Dilated Cardiomyopathy: POSEIDON-DCM Trial. *J Am Coll Cardiol*.

[18] Patel AN, Henry TD, Quyyumi AA, Schaer GL, Anderson RD, Toma C, East C, Remmers AE, Goodrich J (2016). Ixmyelocel-T for patients with ischaemic heart failure: a prospective randomised double-blind trial. *Lancet*.

[19] Bartunek J, Terzic A, Davison BA (2017). Cardiopoietic cell therapy for advanced ischemic heart failure: results at 39 weeks of the prospective, randomized, double blind, sham-controlled CHART-1 clinical trial. *Eur Heart J*.

[20] Mathiasen AB, Qayyum AA, Jørgensen E, Helqvist S, Fischer-Nielsen A, Kofoed KF, Haack-Sørensen M, Ekblond A, Kastrup J (2015). Bone marrow-derived mesenchymal stromal cell treatment in patients with severe ischaemic heart failure: a randomized placebo-controlled trial (MSC-HF trial). *Eur Heart J*.

[21] Perin EC, Silva GV, Henry TD, Cabreira-Hansen MG, Moore WH, Coulter SA (2011). A randomized study of transendocardial injection of autologous bone marrow mononuclear cells and cell function analysis in ischemic heart failure (FOCUS-HF). *Am Heart J*.

[22] Bartunek J, Behfar A, Dolatabadi D (2013). Cardiopoietic stem cell therapy in heart failure: the CCURE (Cardiopoietic stem Cell therapy in heart failure) multicenter randomized trial with lineage specified biologics. *J Am Coll Cardiol*.

[23] Lunde K, Solheim S, Aakhus S, Arnesen H, Moum TR, Abdelnoor M (2007). Exercise capacity and quality of life after intracoronary injection of autologous mononuclear bone marrow cells in acute myocardial infarction: results from the Autologous Stem cell Transplantation in Acute Myocardial Infarction (ASTAMI) randomized controlled trial. *Am heart J*.

[24] Heo S, Lennie TA, Okoli C, Moser DK (2009). Quality of life in patients with heart failure: ask the patients. *Heart Lung*.

[25] Bartolucci J, Verdugo FJ, González PL, Larrea RE, Abarzua E, Goset C (2017). Safety and Efficacy of the Intravenous Infusion of Umbilical Cord Mesenchymal Stem Cells in Patients With Heart Failure: A Phase 1/2 Randomized Controlled Trial (RIMECARD Trial [Randomized Clinical Trial of Intravenous Infusion Umbilical Cord Mesenchymal Stem Cells on Cardiopathy]). *Circ Res.*.

[26] Brehm M, Strauer BE (2006). Stem cell therapy in post infarction chronic coronary heart disease. *Nat Clin Pract Cardiovasc Med*.

